# Inpatient Hospices in Germany: Medical Care Situation and Use of Supportive Oncological Therapies for Symptom Control in Tumor Patients

**DOI:** 10.1089/pmr.2022.0026

**Published:** 2022-08-18

**Authors:** Ulrich Kaiser, Ursula Vehling-Kaiser, Ana Hoffmann, Florian Kaiser

**Affiliations:** ^1^ Clinic and Polyclinic for Internal Medicine III, University Hospital Regensburg, Regensburg, Germany.; ^2^ Oncology/Palliative Care Network Landshut, Landshut, Germany.; ^3^ VK&K Studien GbR, Landshut, Germany.; ^4^ Department of Hematology and Medical Oncology, University Medical Center Göttingen, Göttingen, Germany.

**Keywords:** hematologist/oncologist, hospice, supportive-oncology therapy, symptom control, tumor patient

## Abstract

**Background:**

More than 80% of the residents in German hospices suffer from tumor disease. But the administration of supportive-oncological therapies in hospices for symptom control is controversially discussed.

**Objectives:**

This study aims to investigate the care situation of tumor patients in German hospices with regard to medical care and the use of supportive-oncological therapies.

**Methods:**

In February 2019, all hospices in Germany were offered the opportunity to participate in an anonymous online survey on medical and drug care for their tumor patients. The survey was conducted using the online platform SoSci Survey and ended in April 2019. The analysis was descriptive.

**Results:**

Of 202 hospices, 112 responded to the questionnaire. The hospices were distributed nationwide. Most have 8 to 10 places. More than 80% of hospice residents are tumor patients, and the length of stay is usually three to four weeks. Medical care is primarily provided by primary care physicians. While specialized outpatient palliative care is increasingly involved in care, hematologists/oncologists are rarely represented. Supportive-oncological therapies are rarely prescribed, whereas medication for other chronic conditions is often continued. The percentage of supportive-oncological therapies prescribed is higher in hospices with oncology co-care.

**Conclusions:**

Although most hospice residents suffer from malignant disease, co-care by a hematologist/oncologist is rare. Supportive-oncology therapies, particularly for symptom relief, may therefore be rarely used. However, since a small select group of hospice residents may benefit from these therapies, further investigation in this direction should be undertaken.

## Introduction

In Germany, about 33,500 people with advanced and incurable diseases are currently treated in about 200 inpatient adult hospices yearly.^1^ A predominant number (>80%) of palliative-care patients in both inpatient and outpatient settings suffer from an underlying malignant disease.^2–4^ In 2016, Dasch et al demonstrated that with increasing implementation of inpatient hospices, the proportion of cancer patients who died in hospices also significantly increased.^5^ Current data confirm this trend.^6^ In cancer patients, particularly, complications occur at the end of their lives, which may require specific therapy to alleviate symptoms. These include pain (caused e.g., by osteolysis, meningeosis carcinomatosa/leukemia, or plexus infiltration), bleeding, ulcerating wounds, dyspnea, weakness caused by anemia, or neurological symptoms because of hypercalcemia.

The use of supportive oncological therapies (e.g., radiotherapy, antiproliferative therapies, bisphosphonates, or blood products) with the aim of symptom control and improvement of the quality of life can be a sensible approach here: particularly, the use of antiproliferative therapy in an intravenous, oral, or intrathecal form,^3,7–9^ the transfusion of blood products (erythrocyte concentrates and platelet concentrates)^10,11^ or the administration of human albumin and bisphosphonates have already been successfully used here in palliative patients. This situation is similar to radiotherapy, which can significantly benefit patients for symptom control in enhanced pain therapy or in the case of bleeding or ulcerating tumors.^12–14^

However, their use in hospices is not self-evident, is handled differently, and is controversially discussed.^15,16^ While the integration of palliative medicine in tumor patients is increasingly occurring in the early stages of metastasis parallel to oncological therapies,^17,18^ there are often conflicting ideas about the use of supportive oncological tumor therapies at the end of life, i.e., also in hospices, even if these therapeutic measures are exclusively used to control symptoms.^15,19^ A survey conducted as part of the Hospice and Palliative Survey HOPE in 2007 showed that 6.4% of patients in outpatient and inpatient palliative-care facilities received tumor-specific therapy—except for inpatient hospices. No anticancer therapies were realized here.^20^

Simultaneously, inpatient hospices' medical care and administration are not uniform but include different specialist groups (e.g., general practitioners, palliative medicine physicians, and oncologists); however, medical care and administration are already partly accepted in hospices.^21^ In addition, specific therapies, such as the administration of antiproliferative substances, blood transfusions, and radiotherapy, usually require the cooperation of an oncologist, radiotherapist, or palliative medicine physician with oncological experience.

In principle, it is officially permissible to use oncological therapies, for example, chemotherapy or radiotherapy, in German hospices if they serve exclusively symptom control and not the pursuit of living longer.^22^

However, no comprehensive data are available on the actual form of treatment in hospices in Germany and the medical care of tumor patients in hospices.

Thus, this study is intended to investigate the care situation of patients with an underlying malignant disease in German hospices, especially with respect to the use of supportive oncological therapies for symptom control.

## Methods

### Questionnaire and online platform

**FIG. 1.**
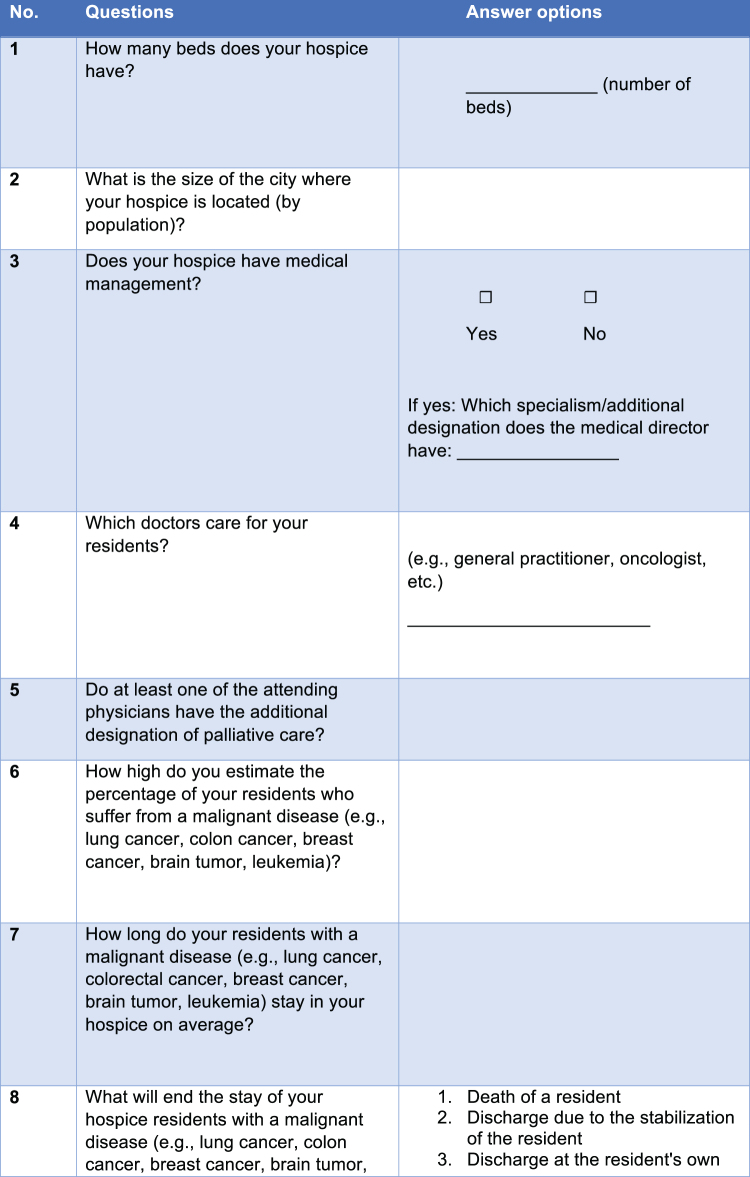

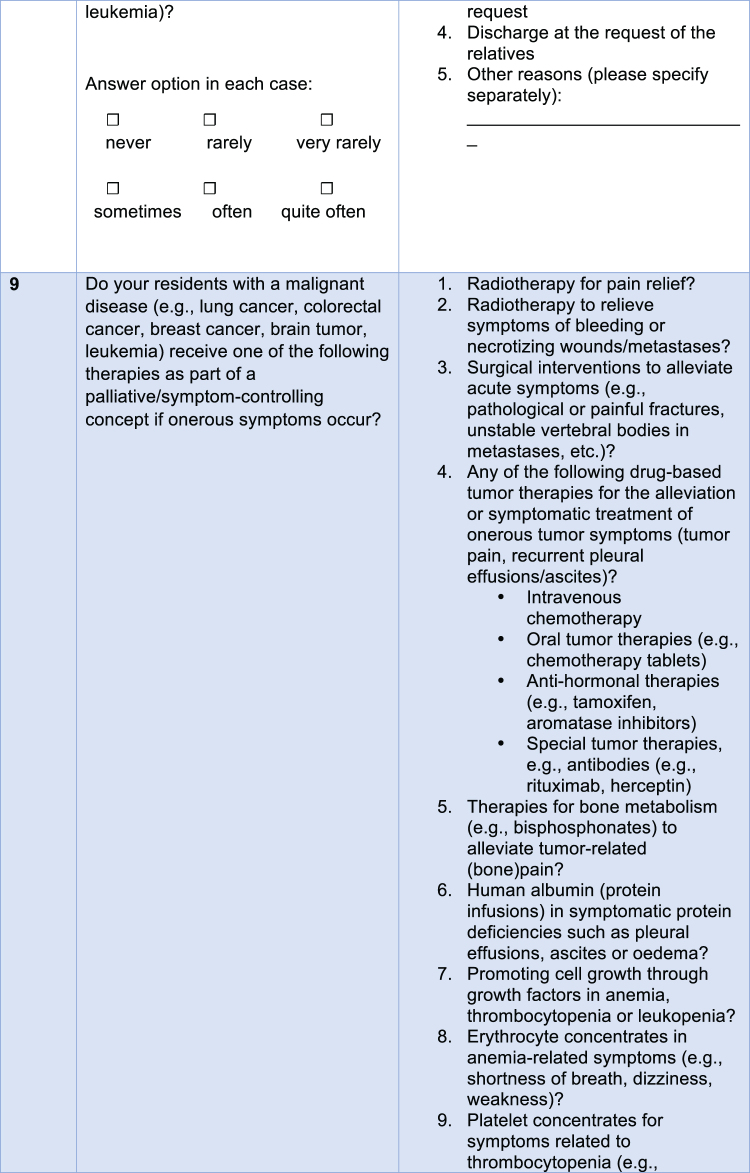

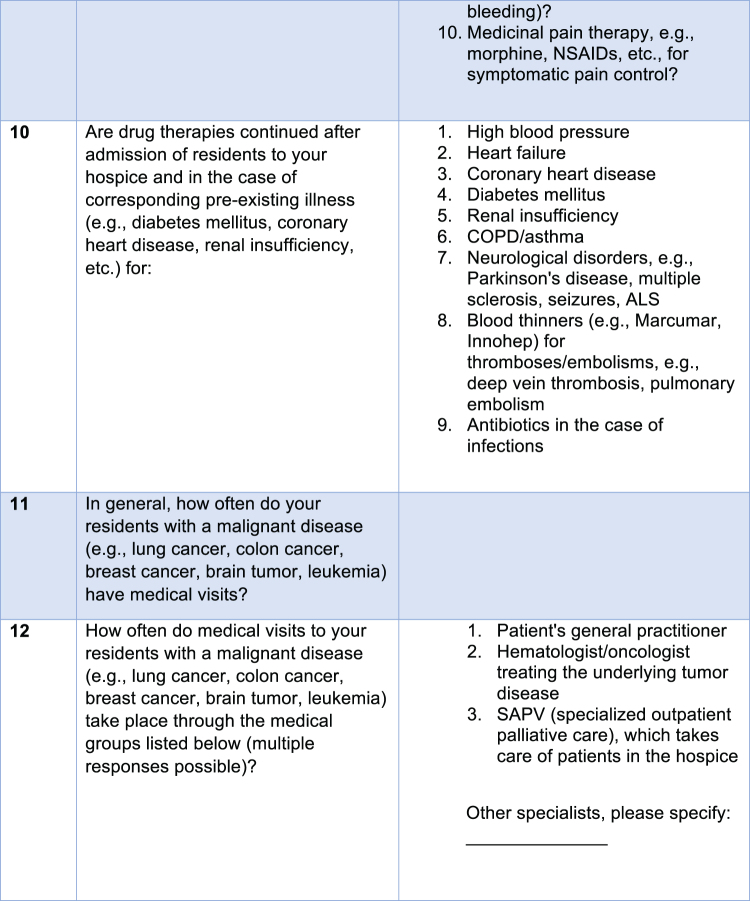
Questionnaire.

The research team developed the questionnaire (Fig. 1) with the cooperation of an expert team of hematologists/oncologists, palliative medicine physicians, general practitioners, and palliative-care nurses, as well as the quality circle for palliative medicine of the cooperating specialized outpatient palliative care (SAPV) team. At the same time, the module “tumor-specific therapy in palliative care” of the Hospice and Palliative Survey HOPE was considered for preparing the questionnaire.^23^ The formal criteria of the questionnaire were based on the GESIS survey guidelines for written questionnaires, the design of rating scales, and online surveys.^24–26^ Before using the questionnaire, an internal and external evaluation was performed for internal and external consistency and comprehensibility of the individual questions. Therefore, experts from the Leibniz Institute for Social Sciences were consulted for this purpose, and a pretest with subsequent feedback among hospice employees was conducted.^27^

The content of the questions related to (1) structural conditions (location, number of beds, diagnoses, and length of stay); (2) medical care (specialist training and management, visits); and (3) the use of different groups of drugs. The questions were asked as a yes/no question (decision question), an open question, or a single choice question using a six-level Likert-scale.^25^ The questionnaire was completed anonymously.

The online platform SoSci Survey, which is freely available for scientific survey projects, was used for the survey. The SoSci platform enables an independent design of the questionnaires. A dynamic design of the questions with different selection types is also possible. We selected either a simple selection option, a drop-down selection, or a multiple selection matrix for this questionnaire. Before the questionnaire was put online, another pretest was conducted through the SoSci platform to test the digital applicability.^26,27^ After the final release, a link to the questionnaire (password-protected) was generated through the platform. After completing the survey, the results were generated automatically in an Excel spreadsheet.

### Hospices interviewed

From February 5 to 14 of 2019, all hospices in Germany (at that time, *n* = 202) were offered the opportunity to participate in an anonymous online survey concerning the medical and medicinal care of tumor patients in hospices. To increase the willingness to participate, each individual hospice was invited to take part personally by telephone.^28,29^ Upon approval, a link with a password to the questionnaire was sent to the respective hospice by e-mail.

To further increase the return of answered questionnaires, reminder e-mails were sent to all participating hospices on March 1 and 29 of 2019 with the request to answer the questionnaire.^28,29^ The questionnaire was accompanied by a short information letter. Reference was made to the high proportion of hospice residents who have cancer. In addition, the aim of the survey was explained, namely, the determination of the role of supportive oncological therapy procedures in the care of hospice residents who have cancer and their medical care in hospices in Germany. The survey period ended on April 26, 2019. Participants were informed about the end of the survey period by e-mail.

### Statistical methods

The evaluation was descriptively conducted. A nominal scale, ordinal scale, and open-ended questions were used as formats for encrypting (encoding) the data. The variant multiple regression was used for the formats.

### Ethical approval

According to the Munich Ethics Committee, no ethical approval was required for this study.

## Results

### Number of participants and questionnaire return

Of the *n* = 202 hospices surveyed, *n* = 11 (5.4%) did not participate in the survey: 2 hospices were not yet open at the time of the survey, and 9 hospices refused to participate. The link to the questionnaire, along with the password, was sent by e-mail to *n* = 191 (94.6%) hospices. A total of 112 (55.4%) hospices answered the questionnaire online. All participating hospices answered >70% of the questions, with 105 hospices answering >85% of all questions.

### Hospice demographics

Most participating hospices (*n* = 86; 76.8%) had 8 to 12 bed spaces (Table 1). The responding hospices were distributed fairly homogeneously over cities of varying population sizes (<20,000 to >500,000) (Table 1).

**Table 1.** Care Data and Demographic Data Hospices

Specialist care hospices, ***n*** (%)	
Does your hospice have a medical management?	
No	100 (89.3)
Yes	12 (10.7)
Specialization of medical director	
Palliative care	4 (3.6)
Hematology and oncology+palliative medicine	3 (2.7)
Psychiatry	2 (1.8)
Internal medicine+palliative care	1 (0.9)
Anesthesia+palliative care	1 (0.9)
General medicine+palliative care	1 (0.9)
Which doctors care for your residents? (Multiple entries possible)	
Physicians without a specified additional designation in palliative medicine	
Family doctor/practitioner/general medicine	65 (58)
Additional specialties if required	23 (20.5)
Oncology	17 (15.2)
Internal medicine	6 (5.4)
Pain medicine	5 (4.5)
Urology	5 (4.5)
Anesthesia	3 (2.7)
Dermatology	2 (1.8)
Dentistry	2 (1.8)
Psychiatry	2 (1.8)
Gynecology	1 (0.9)
Nephrology	1 (0.9)
Neurology	1 (0.9)
Physicians with specified additional designation palliative medicine	
Palliative medicine	65 (58)
SAPV (specialized outpatient palliative care)	24 (21.4)
Anesthesia and palliative care	5 (4.5)
Does at least one of the attending physicians have the additional designation palliative care?	
Yes	112 (100)
No	0 (0)

Medical care hospices, ***n*** (%)	
How often do your residents with a malignant disease generally have medical visits?	
Daily	30 (26.8)
Weekly	81 (72.3)
Every two weeks	1 (0.9)
How often do your residents with a malignant disease generally have medical visits by the physician groups listed below?	
Family doctor	
Daily	6 (5.4)
Weekly	62 (55.4)
Every two weeks	2 (1.8)
Every three weeks	3 (2.7)
Monthly	4 (3.6)
Never	32 (28.6)
Not specified	3 (2.7)
Hematologist/oncologist	
Weekly	10 (8.9)
Every two weeks	3 (2.7)
Monthly	2 (1.8)
Never	91 (81.3)
Not specified	6 (5.4)
SAPV (specialized outpatient palliative care)	
Daily	20 (17.9)
Weekly	54 (48.2)
Every two weeks	2 (1.8)
Never	28 (25)
Not specified	8 (7.1)
Other specialties on demand	30 (26.8)

Termination of hospice stay	
What will end your resident's stay with a malignancy in your hospice? (Multiple entries possible)	
Death of a resident	
Quite often	112 (100)
Discharge due to stabilization of the resident	
Never	4 (3.6)
Very rarely	83 (74.1)
Rarely	19 (17)
Sometimes	6 (5.4)
Discharge at the resident's own request	
Very rarely	108 (96.4)
Rarely	2 (1.8)
Sometimes	2 (1.8)
Discharge due to the wish of the relatives	
Never	66 (58.9)
Very rarely	43 (38.4)
Rarely	2 (1.8)
Sometimes	1 (0.9)
Other reasons	
Rejection cost absorption	4 (3.6)
No hospice indication	4 (3.6)
Hospital admission	2 (1.8)

Demographics	
Percentage of residents suffering from malignant disease	
31%–40%	1 (0.9)
51%–60%	1 (0.9)
61%–70%	1 (0.9)
71%–80%	4 (3.6)
81%–90%	43 (38.4)
91%–100%	62 (55.4)
No. of hospices in correlation to the no. of inhabitants	
Up to 20.000 inhabitants	26 (23.2)
Up to 50.000 inhabitants	21 (18.8)
Up to 100.000 inhabitants	17 (15.2)
Up to 500.000 inhabitants	25 (22.3)
>500.000 inhabitants	22 (19.6)
Not answered	1 (0.9)
Residents' length of stay	
One to two weeks	11 (9.8)
Three to four weeks	86 (71.4)
Five to six weeks	10 (8.9)
Seven to eight weeks	3 (2.7)
Nine to 10 weeks	1 (0.9)
More than 10 weeks	1 (0.9)
Hospice bed spaces	
One bed space	1 (0.9)
Two bed spaces	1 (0.9)
Seven bed spaces	5 (4.5)
Eight bed spaces	25 (22.3)
Nine bed spaces	4 (3.6)
10 bed spaces	36 (32.1)
11 bed spaces	4 (3.6)
12 bed spaces	17 (15.2)
13 bed spaces	3 (2.7)
14 bed spaces	5 (4.5)
16 bed spaces	11 (9.8)

For most participating hospices (*n* = 105; 93.8%), most residents (81%–100%) suffer from a malignant disease. In most hospices (*n* = 86; 76.8%), the average length of stay is three to four weeks; only a small minority of hospices (*n* = 15; 13.4%) have a length of stay of five weeks or longer (Table 1). In all participating hospices (*n* = 112; 100%), most residents die in the hospice. Discharges from the hospice due to stabilization of the patients or at the request of relatives/the patients themselves are relatively rare overall (Table 1). Other reasons for terminating the hospice stay are rare overall, with a lack of funds (*n* = 4; 3.6%) being the most frequently mentioned (Table 1).

### Medical care for hospice residents

Twelve hospices (10.7%) have a medical director, which, in most cases (*n* = 10; 8.9%), has attended further education for palliative medicine. Medical hospice direction by hematologists/oncologists is rare (*n* = 3; 2.7%) (Table 1). All participating hospices (*n* = 112; 100%) generally provide palliative care-oriented medical care (Table 1). Hospice residents are most often treated by general practitioners and/or palliative medicine physicians (*n* = 65; 58% in each case). Care provided by oncologists (*n* = 17; 15.2%) or an SAPV team (*n* = 24; 21.4%) takes place rather less frequently (Table 1). In 23 hospices (20.5%), additional disciplines, such as urology, dentistry, or neurology, are consulted as required (Table 1).

Medical visits are predominantly made on a weekly basis (*n* = 81; 72.3%) to patients with an underlying malignant disease in the participating hospices. In most hospices (*n* = 91; 81.3%), no visits are made by a hematologist/oncologist. In only 15 (13.4%) hospices do hematologist/oncologists visit tumor patients; in 10 (8.9%) hospices these visits occur weekly (Table 1).

### Supportive oncological therapies

Radiotherapy and surgical interventions to alleviate symptoms are never or only very rarely used in most surveyed hospices. If used, the indication for radiotherapy focuses on pain relief more than bleeding or necrotizing wounds (Table 2).

**Table 2.** Use of Supportive Oncological Tumor Therapies for Symptom Relief

	Never, ***n*** (%)	Very rare, ***n*** (%)	Rare, ***n*** (%)	Sometimes, ***n*** (%)	Often, ***n*** (%)	Quite often, ***n*** (%)	Not selected, ***n*** (%)	Total, ***N*** (%)
Medical pain therapy	0 (0)	0 (0)	0 (0)	0 (0)	0 (0)	109 (97.3)	3 (2.7)	112 (100)
Bisphosphonates	19 (20)	37 (33)	22 (19.6)	21 (18.8)	7 (6.3)	0 (0)	6 (5.4)	112 (100)
Erythrocyte concentrates	48 (42.9)	36 (32.1)	12 (10.7)	10 (8.9)	1 (0.9)	0 (0)	5 (4.5)	112 (100)
Antihormonal therapy	16 (14.3)	60 (53.6)	15 (13.4)	13 (11.6)	2 (1.8)	0 (0)	6 (5.4)	112 (100)
Oral tumor therapy	21 (18.8)	66 (58.9)	12 (10.7)	8 (7.1)	0 (0)	0 (0)	5 (4.5)	112 (100)
Radiotherapy for pain relief	49 (43.8)	36 (32.1)	12 (10.7)	12 (10.7)	0 (0)	0 (0)	3 (2.7)	112 (100)
Surgical intervention	46 (41.1)	51 (45.5)	8 (7.1)	4 (3.6)	0 (0)	0 (0)	3 (2.7)	112 (100)
Antibody therapy	52 (46.4)	43 (38.4)	10 (8.9)	2 (1.8)	0 (0)	0 (0)	5 (4.5)	112 (100)
Human albumin	66 (58.9)	28 (25)	9 (8)	4 (3.6)	0 (0)	0 (0)	5 (4.5)	112 (100)
Platelet concentrates	68 (60.7)	30 (26.8)	6 (5.4)	2 (1.8)	0 (0)	0 (0)	6 (5.4)	112 (100)
Intravenous chemotherapy	71 (63.4)	28 (25)	4 (3.6)	6 (5.4)	0 (0)	0 (0)	3 (2.7)	112 (100)
Radiotherapy for bleeding wounds	74 (66.1)	26 (23.2)	4 (3.6)	4 (3.6)	0 (0)	0 (0)	4 (3.6)	112 (100)
Growth factors	78 (69.6)	24 (21.4)	3 (2.7)	1 (0.9)	0 (0)	0 (0)	6 (5.4)	112 (100)

Systemic antiproliferative tumor therapies (intravenous chemotherapy, oral tumor therapy, antihormonal therapy, and antibody therapy) to alleviate symptoms are rarely or never used in most hospices surveyed. However, in a small but relevant proportion of participating hospices (1.8%–11.6%) these therapies are used under the special indication of “alleviation of symptoms” (Table 2).

Systemic nonantiproliferative tumor therapies (bisphosphonates, human albumin, and growth factors) to alleviate symptoms are used in the hospices surveyed, for the most part, rarely or never. An exception is the use of bisphosphonates for pain relief in skeletal metastases, which are used relatively frequently (Table 2).

Blood products (erythrocyte concentrates, platelet concentrates) for reducing tumor-related symptoms are also used very rarely, or not at all, in most hospices. However, there is also a small group of hospices, where blood products (particularly erythrocyte concentrates) are regularly used for symptom control (Table 2).

The most common supportive oncological tumor therapy for treating tumor-related symptoms in hospices is pain therapy, which is used in almost all responding hospices (*n* = 109; 97.3%) (Table 2).

### Continuation of a (nononcological) drug therapy

In most hospices (Table 3), medications for treating common nononcological concomitant diseases (such as high blood pressure, heart failure, coronary heart disease, diabetes mellitus, renal insufficiency, lung diseases (chronic obstructive pulmonary disease [COPD], asthma), neurological diseases, thromboses/embolisms, and infections) are continued regularly and frequently. However, antibiotics for treating infections and blood thinners for thromboses/embolisms tend to be used less often.

**Table 3.** Prescribed Drug Therapy for Nontumor Diseases

	Never, ***n*** (%)	Very rare, ***n*** (%)	Rare, ***n*** (%)	Sometimes, ***n*** (%)	Often, ***n*** (%)	Quite often, ***n*** (%)	Not selected, ***n*** (%)	Total, ***N*** (%)
COPD/asthma	0 (0)	2 (1.8)	1 (0.9)	10 (8.9)	54 (48.2)	42 (37.5)	3 (2.7)	112 (100)
Neurological diseases	1 (0.9)	4 (3.6)	2 (1.8)	15 (13.4)	46 (41.1)	39 (34.8)	5 (4.5)	112 (100)
Diabetes mellitus	0 (0)	1 (0.9)	2 (1.8)	24 (21.4)	46 (41.1)	35 (31.3)	4 (3.6)	112 (100)
Hypertension	1 (0.9)	5 (4.5)	4 (3.6)	35 (31.3)	48 (42.9)	16 (14.3)	3 (2.7)	112 (100)
Heart failure	2 (1.8)	6 (5.4)	4 (3.6)	42 (37.5)	42 (37.5)	13 (11.6)	3 (2.7)	112 (100)
Renal failure	2 (1.8)	6 (5.4)	12 (10.7)	36 (32.1)	41 (36.6)	12 (10.7)	3 (2.7)	112 (100)
Coronary heart disease	1 (0.9)	5 (4.5)	8 (7.1)	43 (38.4)	39 (34.8)	13 (11.6)	3 (2.7)	112 (100)
Anticoagulation	1 (0.9)	17 (15.2)	14 (12.5)	45 (40.2)	27 (24.1)	5 (4.5)	3 (2.7)	112 (100)
Antibiotics	0 (0)	11 (9.8)	14 (12.5)	60 (53.6)	15 (13.4)	9 (8)	3 (2.7)	112 (100)

### Prescription of supportive oncological therapies depending on the medical specialty

The prescription of supportive oncological tumor therapies for symptom control in hospices is performed more frequently in percentage terms by specialists in hematology and oncology. This applies to both antiproliferative and nonantiproliferative medications. In this context, oral medication (antihormonal therapy and oral tumor therapy) predominates in the case of antiproliferative therapies, and the use of bisphosphonates predominates in the case of nonantiproliferative therapies. This trend continues among nononcology specialists for whom antihormonal therapies, in addition to bisphosphonates, are in the foreground (Tables 4 and 5).

**Table 4.** Percentage of the Prescribing Specialist Group (Hospices with Oncologists/Hospices with Nononcologists) in Relation to the Prescription of Antiproliferative Substances

Therapy	Frequency of use	Nononcologists (***N*** = 95), % (***n***)	Oncologists (***N*** = 17), % (***n***)	Total (***N*** = 112), % (***n***)
Oral tumor therapy	Never	20 ( 19)	11.8 ( 2)	18.8 ( 21)
	Very rare	60 ( 57)	52.9 ( 9)	58.9 ( 66)
	Rare	10.5 ( 10)	11.8 ( 2)	10.7 ( 12)
	Sometimes	5.3 ( 5)	17.7 ( 3)	7.1 ( 8)
	Often	0 ( 0)	0 ( 0)	0 ( 0)
	Quite often	0 ( 0)	0 ( 0)	0 ( 0)
	Not selected	4.2 ( 4)	5.9 ( 1)	4.5 ( 5)
Antihormonal therapy	Never	13.7 ( 13)	17.7 ( 3)	14.3 ( 16)
	Very rare	57.9 ( 55)	29.4 ( 5)	53.6 ( 60)
	Rare	10.5 ( 10)	29.4 ( 5)	13.4 ( 15)
	Sometimes	11.6 ( 11)	11.8 ( 2)	11.6 ( 13)
	Often	1.1 ( 1)	5.9 ( 1)	1.8 ( 2)
	Quite often	0 ( 0)	0 ( 0)	0 ( 0)
	Not selected	5.3 ( 5)	5.9 ( 1)	5.4 ( 6)
Antibodies	Never	47.4 ( 45)	41.2 ( 7)	46.4 ( 52)
	Very rare	41.1 ( 39)	23.5 ( 4)	38.4 ( 43)
	Rare	7.4 ( 7)	17.7 ( 3)	8.9 ( 10)
	Sometimes	0 ( 0)	11.8 ( 2)	1.8 ( 2)
	Often	0 ( 0)	0 ( 0)	0 ( 0)
	Quite often	0 ( 0)	0 ( 0)	0 ( 0)
	Not selected	4.2 ( 4)	5.9 ( 1)	4.5 ( 5)
Intravenous chemotherapy	Never	67.4 ( 64)	41.2 (7)	63.4 ( 71)
	Very rare	23.2 ( 22)	35.3 (6)	25 ( 28)
	Rare	3.2 ( 3)	5.9 (1)	3.6 ( 4)
	Sometimes	4.2 ( 4)	11.8 (2)	5.4 ( 6)
	Often	0 ( 0)	0 (0)	0 ( 0)
	Quite often	0 ( 0)	0 (0)	0 ( 0)
	Not selected	2.1 ( 2)	5.9 (1)	2.7 ( 3)

**Table 5.** Percentage of the Prescribing Specialist Group (Hospices with Oncologists/Hospices with Nononcologists) in Relation to the Prescription of Blood Products, Bisphosphonates, and Human Albumin

Therapy	Frequency of use	Nononcologists (***N*** = 95), % (***n***)	Oncologists (***N*** = 17), % (***n***)	Total (***N*** = 112), % (***n***)
Bisphosphonates	Never	17.9 ( 17)	11.8 ( 2)	17 ( 19)
	Very rare	34.7 ( 33)	23.5 ( 4)	33.1 ( 37)
	Rare	21.1 ( 20)	11.8 ( 2)	19.6 ( 22)
	Sometimes	14.7 ( 14)	41.2 ( 7)	18.8 ( 21)
	Often	6.3 ( 6)	5.9 ( 1)	6.3 ( 7)
	Quite often	0 ( 0)	0 ( 0)	0 ( 0)
	Not selected	5.3 ( 5)	5.9 ( 1)	5.4 ( 6)
Human albumin	Never	62.1 ( 59)	41.2 ( 7)	58.9 ( 66)
	Very rare	25.3 ( 24)	23.5 ( 4)	25 ( 28)
	Rare	7.4 ( 7)	11.8 ( 2)	8 ( 9)
	Sometimes	1.1 ( 1)	17.7 ( 3)	3.6 ( 4)
	Often	0 ( 0)	0 ( 0)	0 ( 0)
	Quite often	0 ( 0)	0 ( 0)	0 ( 0)
	Not selected	4.2 ( 4)	5.9 ( 1)	4.5 ( 5)
Erythrocyte concentrates	Never	45.3 ( 43)	29.4 ( 5)	42.9 ( 48)
	Very rare	33.7 ( 32)	23.5 ( 4)	32.1 ( 36)
	Rare	9.5 ( 9)	17.7 ( 3)	10.7 ( 12)
	Sometimes	7.4 ( 7)	17.7 ( 3)	8.9 ( 10)
	Often	0 ( 0)	5.9 ( 1)	0.9 ( 1)
	Quite often	0 ( 0)	0 ( 0)	0 ( 0)
	Not selected	4.2 ( 4)	5.9 ( 1)	4.5 ( 5)
Platelet concentrates	Never	63.2 ( 60)	47.1 ( 8)	60.7 ( 68)
	Very rare	26.3 ( 25)	29.4 ( 5)	26.8 ( 30)
	Rare	4.2 ( 4)	11.8 ( 2)	5.4 ( 6)
	Sometimes	1.1 ( 1)	5.9 ( 1)	1.8 ( 2)
	Often	0 ( 0)	0 ( 0)	0 ( 0)
	Quite often	0 ( 0)	0 ( 0)	0 ( 0)
	Not selected	5.3 ( 5)	5.9 ( 1)	5.4 ( 6)

## Discussion

The care of tumor patients in hospices still represents an area of tension in the field of palliative medicine.^3^ However, tumor-specific therapies aimed at life extension in the last phase of life are regarded as a burden, and their termination is demanded.^30^ On the other hand, selected therapies can significantly alleviate symptoms in certain patients.^10,12,20,31^ With the increasing number of hospices in Germany, the proportion of patients suffering from malignant disease and spending their last stage of life in hospice increases accordingly. Currently, about 26,800 tumor patients spend the last phase of their lives in inpatient hospices.^1,2^ This study investigated the medical care of these patients.

Since the responding hospices (1) were evenly distributed across Germany, that is, urban and rural areas were both represented (Table 1); (2) had similar bed capacities (Table 1), and (3) the response rate was 55% (participation of 112 out of 202 requested hospices), thus meaningful generalizable data were generated.^32–34^

Most hospices in Germany are supervised by general practitioners with or without additional training in the field of palliative medicine. In a smaller proportion, consultants from various disciplines and/or an SAPV team are involved. At this time, hematologists/oncologists are rarely represented. In principle, a palliative medical care option is ensured for all hospices (Table 1). These data are consistent with other surveys concerning medical hospice care.^21^

As in palliative care more generally,^4^ most hospice residents suffer from malignant diseases (Table 1). In most of the hospices surveyed, the patient's stay is three to four weeks, however, a smaller proportion of the residents seem to remain in the hospice for several weeks to months (Table 1). Especially for this patient group with an even longer lifetime, the question arises in the literature concerning the use of a supportive oncological therapy to alleviate symptoms.^3^ For these therapies, feasibility and practicability in hospices^3,7^ and a positive influence on the quality of life of palliative patients has been demonstrated.^12–14,35^ Our study confirmed the use of supportive oncological therapies in inpatient hospices. But the future challenge will be the identification of suitable patients in hospices. Further investigations are certainly required.^3^

However, the results of the current survey provide indications that supportive oncological therapies are used only in a small minority of hospices in Germany or are only rarely included in the therapy of severely ill patients with underlying malignant disease (Table 2). Since the proportion of tumor patients in the hospices is comparable, this could either be due to a lack of fundamental consensus on the use of supportive oncological therapies in hospices^10,12,20,30,31^ and/or to a lack of care: On the one hand, only a small proportion of hospices are co-supported by a hematologist/oncologist (Table 1) and, on the other hand, the use of supportive oncological tumor therapies in tumor patients in hospices seems to be influenced by the co-supervision by a hematologist/oncologist; in hospices with hematologists/oncologists, these therapies were used more frequently in percentage terms than in hospices without hematologists/oncologists (Tables 4 and 5).^3^

This could be partly due to the fact that general practitioners—who are responsible for most of the medical care in hospices (Table 1)—usually prefer the use of tumor-specific therapies to be performed by their respective specialists.^36^ This is also supported by the fact that, of the supportive oncological tumor therapies, bisphosphonates—which may not be associated with the special tumor therapeutics in the view of the prescribing physicians—are most frequently prescribed by nononcologists for treating tumor-related bone pain.

In contrast to supportive oncological therapies, existing medications for chronic diseases, such as COPD, heart failure, or diabetes mellitus, are often continued in hospices (Table 3). The continued use of such chronic medication in palliative medicine and especially in hospices is controversially discussed.^37,38^ In the case of diabetes mellitus, an elevated blood sugar is often tolerated to avoid hypoglycemia, and the recommendations of the specialist society are more in favor of a reduction of the antidiabetics.^39^ For treating the symptoms of dyspnea, which is frequently described especially in cardiological and pneumological patients,^40,41^ opiates are often used before other medications.^41,42^ Especially toward the end of life, the symptoms of fatigue, lack of appetite, and weakness also come to the fore in nononcological diseases.^42^ The continued use of chronic medication in inpatient hospices will certainly have to be discussed critically with regard to whether they should be continued when their benefits are typically realized only after years.

Overall, the use of nononcological (chronic) medication and supportive oncological tumor therapy in hospices seems to be influenced by the medical discipline in charge. Another limiting factor could be structural conditions, such as those required for special supportive oncological therapies (e.g., radiotherapies or transfusion of blood products).^3^

Taking into account the increasing establishment of day hospices in Germany^43,44^ and the variable lifetime of hospice residents, the use of supportive oncological therapy options in the hospice sector should always be carefully examined to achieve the best possible symptom control and thus the best possible quality of life for patients.

### Limitations

There is only information available from just over half of German hospices. However, this response rate is considered sufficient, especially in online surveys, to make valid statements.^32–34^ With regard to subject-specific care, especially the number of supervising hematologists/oncologists [*n* = 17 (15.2%) in all participating hospices] may be underestimated, as increasing SAPV care may involve hematology/oncology-trained SAPV physicians in the care of hospice patients. In addition, the care provided by palliative-care physicians, as indicated by the hospices, does not allow conclusions to be drawn about the basic specialism of the corresponding physicians, limiting statements about specialist medical care. It was not possible to identify the representative from each hospice who answered the survey without fail. Therefore, different perspectives from individual disciplines (nurses, physicians, administrators, etc.), which may have influence on the response behavior, cannot be excluded.

## Conclusions

General practitioners with or without additional palliative medicine qualifications usually provide care for patients with an underlying malignant disease in hospices. Co-treatment by a hematologist/oncologist is rare but is nevertheless practiced in a small minority of hospices. In contrast to nononcological medications, supportive oncological therapies are never or rarely used. However, if a hematologist/oncologist also supervises a hospice, supportive oncological therapies are more frequent in percentage terms.

At this time of care, it is reasonable to assume that hematology/oncology and palliative medicine are only occasionally practiced as a common care concept in the sense of holistic care of tumor patients in hospices in Germany.^45^ As already implemented in some hospices, hospice care and the administration of supportive oncological therapies for improved symptom control can be a common and feasible care concept for tumor patients in the last stage of their disease.^46^ There are, of course, a variety of open questions/obstacles to increased use, such as assumption of costs, training of physicians, optimal patient selection and overall assessment of the benefits versus burdens of supportive oncological interventions.

## Abbreviations Used

COPD: chronic obstructive pulmonary disease
SAPV: specialized outpatient palliative care
